# Monitoring Microcirculatory Blood Flow with a New Sublingual Tonometer in a Porcine Model of Hemorrhagic Shock

**DOI:** 10.1155/2015/847152

**Published:** 2015-10-04

**Authors:** Péter Palágyi, József Kaszaki, Andrea Rostás, Dániel Érces, Márton Németh, Mihály Boros, Zsolt Molnár

**Affiliations:** ^1^Department of Anesthesiology and Intensive Therapy, University of Szeged, 6 Semmelweis Street, Szeged 6725, Hungary; ^2^Institute of Surgical Research, University of Szeged, 6 Szőkefalvi-Nagy Béla Street, Szeged 6720, Hungary

## Abstract

Tissue capnometry may be suitable for the indirect evaluation of regional hypoperfusion. We tested the performance of a new sublingual capillary tonometer in experimental hemorrhage. Thirty-six anesthetized, ventilated mini pigs were divided into sham-operated (*n* = 9) and shock groups (*n* = 27). Hemorrhagic shock was induced by reducing mean arterial pressure (MAP) to 40 mmHg for 60 min, after which fluid resuscitation started aiming to increase MAP to 75% of the baseline value (60–180 min). Sublingual carbon-dioxide partial pressure was measured by tonometry, using a specially coiled silicone rubber tube. Mucosal red blood cell velocity (RBCV) and capillary perfusion rate (CPR) were assessed by orthogonal polarization spectral (OPS) imaging. In the 60 min shock phase a significant drop in cardiac index was accompanied by reduction in sublingual RBCV and CPR and significant increase in the sublingual mucosal-to-arterial PCO_2_ gap (P_SL_CO_2_ gap), which significantly improved during the 120 min resuscitation phase. There was significant correlation between P_SL_CO_2_ gap and sublingual RBCV (*r* = −0.65, *p* < 0.0001), CPR (*r* = −0.64, *p* < 0.0001), central venous oxygen saturation (*r* = −0.50, *p* < 0.0001), and central venous-to-arterial PCO_2_ difference (*r* = 0.62, *p* < 0.0001). This new sublingual tonometer may be an appropriate tool for the indirect evaluation of circulatory changes in shock.

## 1. Introduction

Disturbances of the microcirculation are tightly linked to circulatory failure of different origin; thus evaluation of the microcirculatory status has gained increasing importance in the diagnosis and treatment of critically ill patients. It is recognized that in spite of the normal values of global oxygen delivery regional tissue hypoperfusion may exist, which cannot be detected by conventional monitoring tools [1, 2]. Besides, compensatory mechanisms may lead to the normalization of macrohemodynamic parameters in the early phase of circulatory shock, and silently ongoing microcirculatory insufficiencies can cause cellular hypoxia and metabolic dysfunctions, eventually leading to organ failure [3].

The measurement of the partial pressure of carbon dioxide (PCO_2_) in tissues is a potentially feasible technique for the indirect evaluation of the microcirculation [4, 5]. This parameter reflects the adequacy of regional microvascular blood flow, as intramucosal PCO_2_ is inversely related to the proportion of well perfused capillaries, and is mainly dependent on tissue perfusion [6, 7]. However, acute increases or decreases in the PCO_2_ of the arterial blood (P_a_CO_2_) result in comparable changes in the tissue PCO_2_ [8]; thus it should be interpreted in relation to P_a_CO_2_. By subtracting P_a_CO_2_ from the tissue PCO_2_, special gap values can be calculated which are more accurate than the mucosal PCO_2_ alone, as they are independent of concurrent changes in P_a_CO_2_. Although there is no consensus on the most sensitive hemodynamic and laboratory parameters indicating the onset of shock, the tissue-to-arterial PCO_2_ gap may provide an early and important additional signal of perfusion failure [1, 2, 9]. In addition, the tissue-to-arterial PCO_2_ gap values also have prognostic importance [1, 10, 11]; therefore the monitoring of tissue levels of CO_2_ may be helpful in titrating therapeutic interventions in critical states [12], or in selecting patients with compromised physiologic reserve who require expanded hemodynamic monitoring.

Different sites of the gastrointestinal tract are available for the purpose of tissue capnometry and the assessment of the adequacy of mucosal blood flow. As PCO_2_ results gained from the stomach and the sublingual regions proved to be interchangeable [6, 13], and the latter is free of some limitations of gastric tonometry, such as interference of gastric acid, enteral feeding, and potential pitfalls of pHi calculation [14], sublingual tonometry may be a useful alternative for measuring mucosal PCO_2_. Though promising, this technique is still not available at the bedside because of the lack of a suitable monitoring device; hence clinical and experimental evidence on its efficacy is also missing.

**Figure 1:**
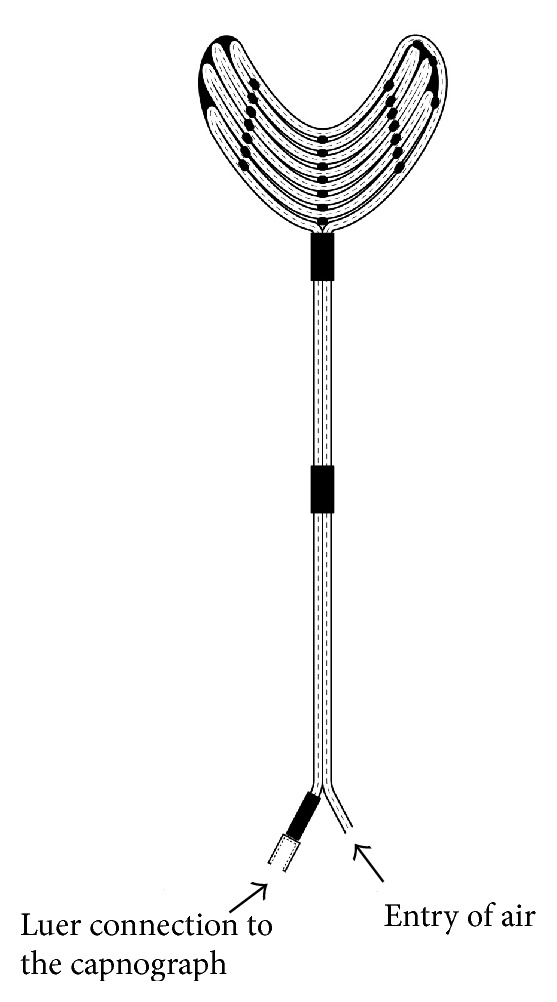
The new capillary tonometer. Illustration of the new sublingual tonometer applied during the examinations.

It is generally acknowledged that monitoring of the sublingual microcirculation, the only site of intravital microscopy (IVM) available at the point of care for most critically ill patients, is of particular prognostic value [3]. In our institute a special instrument has been designed and manufactured for the measurement of sublingual PCO_2_ (Figure 1), which is a further development of a gastric tonometer [15, 16]. The performance of this new probe was recently tested* in vitro* and also in patients with respiratory disease, and the results showed its suitability for sublingual tonometry [17].

The main goal of the current study was to test this new sublingual probe in a porcine model of hemorrhagic shock and compare its performance to direct microcirculatory measurements with IVM using the orthogonal polarization spectral (OPS) imaging technique. Another aim was to investigate how the capnometry-derived values relate to global indicators of hemodynamic changes during hemorrhage and resuscitation. We also hypothesized that if the same diagnostic end points can be reached, sublingual capnometry could offer a technically simpler, alternative method to monitor sublingual microcirculatory changes noninvasively.

## 2. Materials and Methods

The experiments were carried out in strict adherence to the National Institute of Health guidelines for the use of experimental animals and the study was approved by the Ethics Committee and the Institutional Animal Care and Use Committee at the University of Szeged. The study was conducted in the research laboratory of the Institute of Surgical Research in a manner that does not inflict unnecessary pain or discomfort upon the animals.

### 2.1. Animals and Instrumentation

Thirty-six Vietnamese mini pigs of both genders, weighing 16–25 kg, underwent a 24 hr fasting preoperatively with free access to water; the animals were randomly allocated into control (sham-operated, *n* = 9) and hemorrhagic shock groups (shock, *n* = 27). Anesthesia was induced by an intramuscular injection with a mixture of ketamine (20 mg kg^−1^) and xylazine (2 mg kg^−1^) and maintained with a continuous infusion of propofol (50 *μ*g min^−1^ kg^−1^ iv, 3 mg kg^−1^ hr^−1^). After endotracheal intubation, the animals were mechanically ventilated with room air (Harvard Apparatus, South Natick, MA, USA). The tidal volume was set at 9 ± 2 mL kg^−1^, and the respiratory rate was adjusted to maintain the end-tidal partial pressure of carbon dioxide (EtCO_2_) and P_a_CO_2_ in the range of 35–45 Torr (4.7–6.0 Pa). The depth of anesthesia was assessed by monitoring the jaw tone regularly. The animals were placed in supine position on a heating pad for maintenance of the body temperature between 36 and 37°C.

For measurement of the sublingual PCO_2_ (P_SL_CO_2_) the new sublingual capillary tonometer (see below) was placed under the tongue, and a specially designed latex face mask was used to close the oral cavity. Capnography was performed with a Microcap handheld capnograph (Oridion Medical Ltd, Jerusalem, Israel). The sublingual mucosal-to-arterial PCO_2_ difference (P_SL_CO_2_ gap) was calculated by subtracting P_SL_CO_2_ from the simultaneously taken P_a_CO_2_ values.

For central venous access the left jugular vein was catheterized. A three-lumen central venous catheter (7 F, Edwards Lifesciences LLC, Irvine, USA) was introduced for blood sampling and fluid administration using aseptic surgical technique. The central venous pressure (CVP) was monitored continuously with a computerized data-acquisition system (SPELL Haemosys; Experimetria Ltd., Budapest, Hungary). For hemodynamic measurements a special thermodilution catheter (Pulsiocath, PULSION Medical Systems AG, Munich, Germany) was placed into the left femoral artery. The cardiac output was monitored by transpulmonary thermodilution and continuous pulse contour analysis (PiCCO method). The right carotid artery was also catheterised for bleeding (7 F, PE, Access Technologies, Illinois, USA). The blood gas measurements were carried out by taking arterial and central venous blood samples simultaneously according to the study protocol, which were then analyzed by cooximetry with a blood gas analyzer (Cobas b221, Roche, Austria). Simplified oxygen extraction rate (O_2_ER) was calculated according to the standard formula from arterial (SaO_2_) and central venous oxygen saturations (ScvO_2_): O_2_ER = (SaO_2_ − ScvO_2_)/SaO_2_. From the central venous and arterial blood gas values the central venous-to-arterial PCO_2_ gap (PcvaCO_2_) was also determined.

For direct evaluation and noninvasive visualization of the sublingual microcirculation the intravital OPS imaging technique (Cytoscan A/R, Cytometrics, Philadelphia, PA, USA) was used. A 10x objective was placed onto the sublingual mucosa, and microscopic images were recorded with an S-VHS video recorder (Panasonic AG-TL 700, Matsushita Electric Ind. Co. Ltd, Osaka, Japan). Quantitative assessment of the microcirculatory parameters was performed offline by frame-to-frame analysis of the videotaped images. Red blood cell velocity (RBCV; *μ*m s^−1^) changes in the postcapillary venules were determined in three separate fields by means of a computer-assisted image analysis system (IVM Pictron, Budapest, Hungary) [18]. Capillary perfusion rate (CPR; 1/1) was determined as the length of continuously perfused microvessels per total length of capillaries in the observational area. During quantitative assessment of CPR we used a diameter limitation for determination of the microvascular network. Exclusively those vessels were selected for analysis, whose diameters were less than 20 *μ*m. All microcirculatory evaluations were performed by the same investigator.

### 2.2. Description of the New Tonometric Probe

The new sublingual capillary tonometer (Mediszintech Ltd, Budapest, Hungary) is a specially coiled silicone rubber tube (ID: 1.5 mm, OD: 2.0 mm, and length: 640 mm) with high permeability for gases, which is formed into a multiple V-shape by using a mould and is glued along five lines (Figure 1). To prevent the soft-walled tube from flattening, a polyamide fiber of 0.3 mm thickness is inserted along its full length. Thereby after folding the tube a sufficient gap remains ensuring the free transport of the filling medium. The afferent and deferent parts of the tube are fixed together at their branching. The end of the deferent tube is equipped with a Luer connector. The filling material is room air, which equilibrates quickly with the PCO_2_ content of the capillaries in the sublingual mucosa. After the required equilibration time it can be aspirated and measured by capnometry. The duration of the full equilibration of the sublingual probe is about 15 minutes. The PCO_2_ of the aspirated gas is measured by infrared spectrophotometry. The results are immediately displayed in units of mmHg.

### 2.3. Experimental Protocol

**Figure 2:**
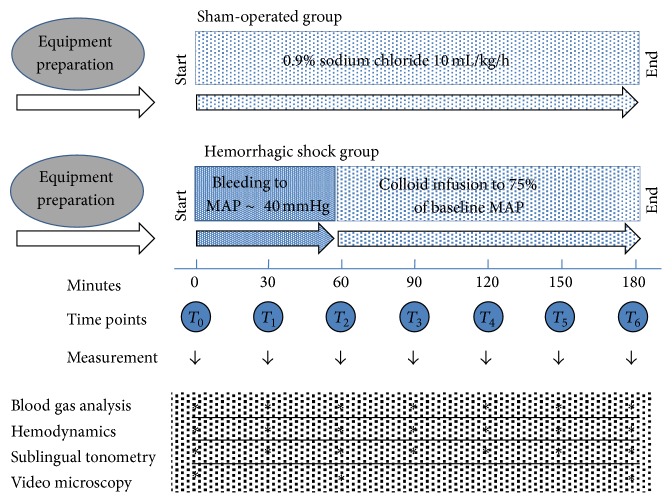
Experimental protocol. Flow diagram representing the experimental protocol in both groups of animals. MAP is mean arterial pressure, *T*_0_–*T*_6_ are seven time points of measurements, and ∗ indicates the implementation of different types of measurements.

The preparation period was followed by a 30 min resting period. After baseline measurements at 0 min (*T*_0_) in the shock group, hemorrhagic shock was induced by bleeding the animals through the right carotid arterial catheter into a heparin (100 IU mL^−1^) containing reservoir. The target mean arterial pressure (MAP) of approximately 40 mmHg was reached in 10–15 min and was kept by repeated bleeding periods until the 60th min of the experiment (*T*_2_). The amount of shed blood was precisely monitored. The average blood loss was about 25 mL kg^−1^ 15 min after the onset of hemorrhage, which increased to an average of around 40 mL kg^−1^ by the end of bleeding at *T*_2_. This was about 50% of the animals' circulating blood volume. At 60 min (*T*_2_) volume resuscitation with colloid solution (hydroxyethyl starch 130 kDa/0.4, 6% Voluven, Fresenius, Germany) was started. 75% of the starting MAP was reached in 10–15 min. In case of decreasing blood pressure further colloid infusion was given, but the total amount of colloid infusion was maximized in 25 mL kg^−1^. This means that the pigs were partially resuscitated and remained hypovolemic in the following period between 60 and 180 min (*T*_2_ and *T*_6_). The reason for choosing this protocol was to enable us to investigate the alterations of different macro- and microcirculatory parameters in two well separated periods: severe shock and moderate hypovolemia. Hemodynamic, arterial, and central venous blood gas measurements and tissue capnometry were repeated and recorded every 30 min for duration of 3 hr (*T*_0_–*T*_6_). Intravital video microscopy was performed at baseline, at 60 (*T*_2_), and at 180 min (*T*_6_) (Figure 2).

Animals in the control group were not submitted to bleeding. They underwent the same operation procedure and received the same instrumentation and monitoring. In this group 0.9% sodium chloride was infused at a rate of 10 mL kg^−1^ h^−1^ during the experiment. Hemodynamic, blood gas analysis and microcirculatory measurements were performed at the same time points.

### 2.4. Statistical Analysis

The statistical software package SigmaStat for Windows (Jandel Scientific, Erkrath, Germany) was applied for data analysis. After testing for normality parametric methods were used. Two-way repeated measures analysis of variance (ANOVA) was applied for statistical analysis. For the analysis of differences between the sham-operated and the hemorrhagic shock groups, the time dependent differences from the baseline (*T*_0_) for each group were assessed by Holm-Sidak post hoc test. When we examined the effect of partial resuscitation starting at 60 minutes (*T*_2_), we performed multiple pairwise comparisons of *T*_3_–*T*_6_ results versus *T*_2_ data serving as control. The pairwise comparison of different variables was made with Pearson-correlation. *p* values < 0.05 were considered statistically significant. The numeric data in the text and values on the figures are given as mean and standard deviations.

## 3. Results

### 3.1. Hemorrhagic Shock Phase (*T*_0_ to *T*_2_)

**Figure 3:**
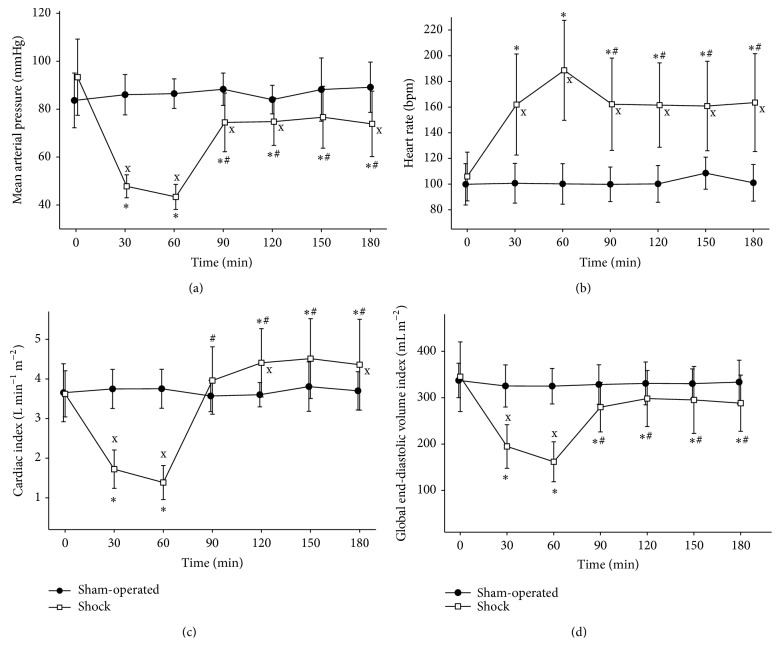
Macrohemodynamic parameters. Changes of macrohemodynamic parameters, mean arterial pressure (a), heart rate (b), cardiac index (c), and global end-diastolic volume index (d). ^∗^ *p* < 0.05 as compared to 0 min (*T*_0_), ^#^ *p* < 0.05 as compared to 60 min (*T*_2_), and ^x^ *p* < 0.05 shock group versus sham-operated group.

**Figure 4:**
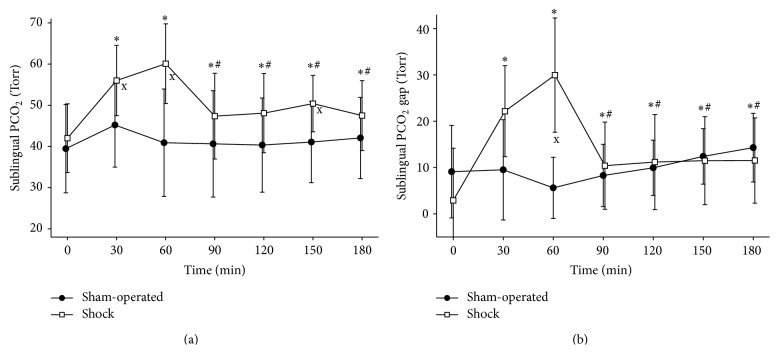
Sublingual capnometry. Changes of sublingual tonometric variables measured by the new probe, sublingual PCO_2_ (a) and sublingual PCO_2_ gap (b). ^∗^ *p* < 0.05 as compared to 0 min (*T*_0_), ^#^ *p* < 0.05 as compared to 60 min (*T*_2_), and ^x^ *p* < 0.05 shock group versus sham-operated group.

**Figure 5:**
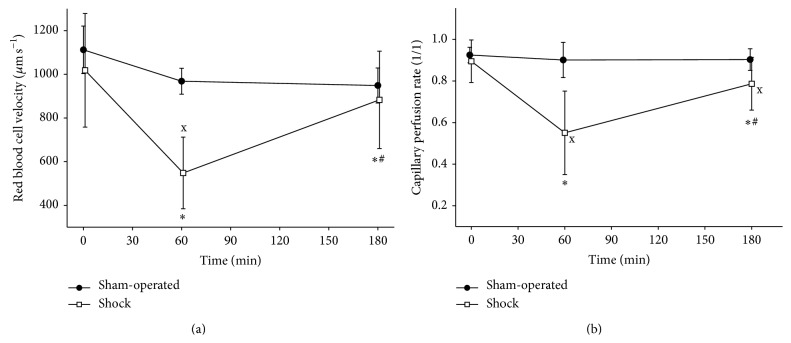
Microcirculatory parameters. Changes of microcirculatory parameters measured by orthogonal polarization spectral imaging, red blood cell velocity in postcapillary venules (a) and capillary perfusion rate (b). ^∗^ *p* < 0.05 as compared to 0 min (*T*_0_), ^#^ *p* < 0.05 as compared to 60 min (*T*_2_), and ^x^ *p* < 0.05 shock group versus sham-operated group.

**Figure 6:**
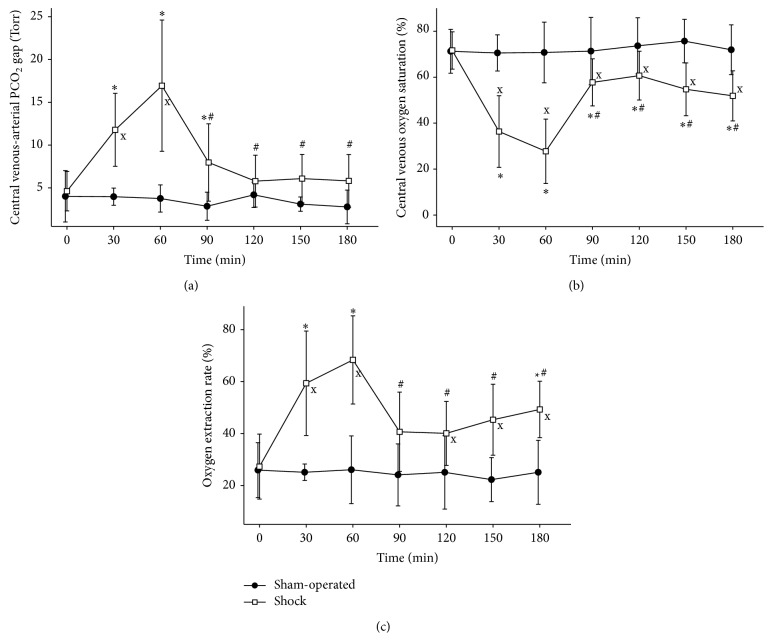
Central venous blood gas derived parameters. Changes of central venous blood derived parameters, central venous-arterial PCO_2_ gap (a), central venous oxygen saturation (b), and oxygen extraction rate (c). ^∗^ *p* < 0.05 as compared to 0 min (*T*_0_), ^#^ *p* < 0.05 as compared to 60 min (*T*_2_), and ^x^ *p* < 0.05 shock group versus sham-operated group.

Severe shock state was achieved in the animals of the shock group as indicated by marked and significant changes in macrohemodynamics during the first 60 minutes: MAP decreased, heart rate (HR) increased, and cardiac index (CI) and global end-diastolic volume index (GEDVI) decreased significantly (Figures 3(a), 3(b), 3(c), and 3(d)). This change in global hemodynamics was accompanied by a significant drop in base excess (BE) in the shock group (*T*_0_: 6.4 ± 2.1 and *T*_2_: 1.1 ± 2.8 mmol L^−1^ *p* < 0.05), while there was no similar change in the sham group (*T*_0_: 5.6 ± 1.8 and *T*_2_: 5.9 ± 1.9 mmol L^−1^). We detected significant increases both in the P_SL_CO_2_ and in the P_SL_CO_2_ gap values (Figures 4(a) and 4(b)). The sublingual postcapillary red blood cell velocity (RBCV_SL_) and the sublingual capillary perfusion rate (CPR_SL_) decreased significantly (Figures 5(a) and 5(b)). The central venous blood derived variables showed characteristic alterations too: corresponding to the significant increase of the oxygen extraction rate the ScvO_2_ decreased during bleeding, while the PcvaCO_2_ increased (Figures 6(a), 6(b), and 6(c)). These changes at 60 minutes were significant compared to the baseline values and differed significantly from the corresponding values of the sham-operated animals.

### 3.2. Partial Resuscitation Phase (*T*_2_ to *T*_6_)

Statistically significant alterations were found regarding MAP, HR, CI, and GEDVI (Figures 3(a), 3(b), 3(c), and 3(d)). The CI increased significantly at *T*_4_, *T*_5_, and *T*_6_ compared to baseline values and at *T*_4_ and *T*_6_ compared to the sham-operated group as well (Figure 3(c)). Improvement in global hemodynamics was also reflected by the significant improvement in BE in the shock group from *T*_2_ to *T*_6_ (1.1 ± 2.8, 3.1 ± 3.4 mmol L^−1^, *p* < 0.05, resp.), while there was no change in BE in the sham group (5.9 ± 1.9, 6.7 ± 2.9 mmol L^−1^). The P_SL_CO_2_ did not change significantly over time in the sham-operated group. In the shock group there was also a significant improvement during this period, still these values remained elevated as compared to baseline (*T*_0_). Moreover, at *T*_5_  P_SL_CO_2_ was significantly higher than in the sham-operated group (Figure 4(a)). Regarding the P_SL_CO_2_ gap, it decreased significantly by *T*_3_ as compared to *T*_2_ in the shock group, but it remained significantly higher as compared to *T*_0_ throughout the resuscitation period. In the sham-operated group the P_SL_CO_2_ gap showed a slow nonsignificant increase over time. Between *T*_3_ and *T*_6_ there were no significant differences between the sham and shock groups (Figure 4(b)).

Concerning the microcirculatory measurements, both RBCV_SL_ and CPR_SL_ increased significantly in the shock group compared to *T*_2_, but still they remained decreased compared to the baseline values. At 180 min (*T*_6_), there was no difference in RBCV_SL_ between shock and sham-operated groups (Figure 5(a)), while CPR_SL_ in the shock group remained significantly lower than in the sham-operated group (Figure 5(b)).

Samples of the pictures in each phase can be seen as electronically submitted Supplementary Material (see Figure S1 available online at http://dx.doi.org/10.1155/2015/847152).

Fluid resuscitation resulted in a significant decrease of the PcvaCO_2_ at *T*_3_–*T*_6_ as compared to *T*_2_, but PcvaCO_2_ changes within the shock group were significant at *T*_3_ compared to *T*_0_ (Figure 6(a)). ScvO_2_ showed a statistically significant elevation after resuscitation as compared to *T*_2_ but remained significantly lower as compared to the baseline value at *T*_0_ and to the sham-operated group (Figure 6(b)). In case of the oxygen extraction rate significant differences were observed between the sham and shock groups at *T*_4_–*T*_6_ (Figure 6(c)).

### 3.3. Correlation Analysis

**Figure 7:**
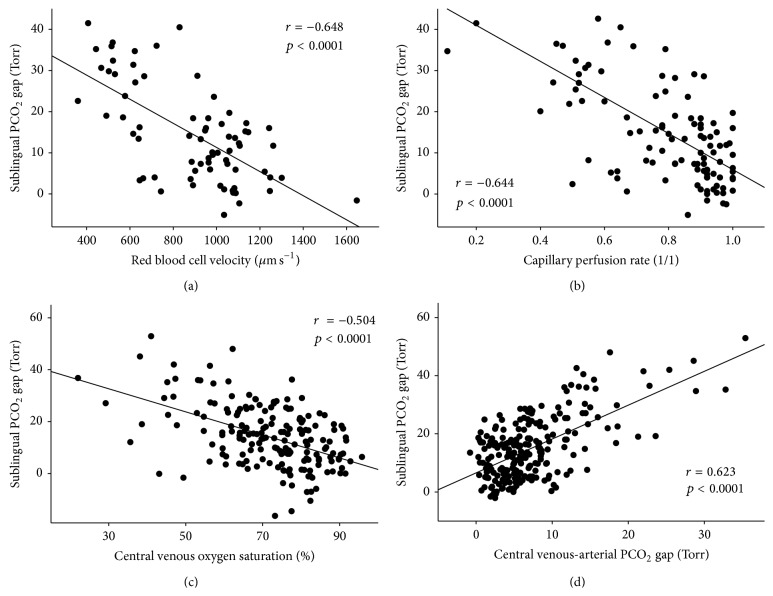
Correlations with sublingual capnometry. Relationships between sublingual mucosal-to-arterial carbon-dioxide partial pressure gap and sublingual red blood cell velocity in postcapillary venules (a), sublingual capillary perfusion rate (b), central venous oxygen saturation (c), and central venous-to-arterial carbon-dioxide partial pressure difference (d).

Statistically significant correlation was found between P_SL_CO_2_ gap and RBCV_SL_ (*r* = −0.648; *p* < 0.0001) and P_SL_CO_2_ gap and CPR_SL_ (*r* = −0.644; *p* < 0.0001) (Figures 7(a) and 7(b)). The P_SL_CO_2_ gap also correlated with ScvO_2_ and PcvaCO_2_ (*r* = −0.504 and *p* < 0.0001; *r* = 0.623 and *p* < 0.0001, resp.) (Figures 7(c) and 7(d)). A significant but weaker correlation was found between P_SL_CO_2_ and ScvO_2_ (*r* = −0.29; *p* < 0.0001) and P_SL_CO_2_ and PcvaCO_2_ (*r* = 0.405; *p* < 0.0001).

## 4. Discussion

In this study we report on the first* in vivo* application of a new sublingual tonometric device. The major finding of this experiment is that this noninvasive monitor accurately followed the changes in submucosal postcapillary blood flow during bleeding and resuscitation. The measured values showed very good correlation with direct indices of microcirculation as determined by the well-established OPS technique and also with global measures of hypovolemia-caused oxygen debt such as ScvO_2_ and PcvaCO_2_.

### 4.1. Sublingual Capnometry and Microcirculation

There are different methods able to detect the increased concentrations of CO_2_ in the periphery. Gastric tonometry is based upon the monitoring of gastric mucosal PCO_2_ level; sublingual and buccal capnometry measure mucosal PCO_2_ of the proximal gastrointestinal tract [19–21]. Mixed venous-to-arterial or central venous-to-arterial CO_2_ partial pressure difference is regarded as markers describing the balance between cardiac output and oxygen consumption by peripheral tissues [22, 23].

The concept of monitoring complementary regional/local perfusion parameters in order to guide or fine-tune resuscitation strategies is rather old and well-established. Historically, one of the first methods was gastric tonometry. However, technical difficulties, long equilibration, and other confounding factors [14] hindered the widespread use of the method, leading to the withdrawal of these devices from the market. In recent years several investigators came to the conclusion that PCO_2_ values of the oral mucosa correlate well with gastric mucosal PCO_2_ parameters [6, 13, 20, 24]. Although the value of sublingual capnometry in the diagnosis of circulatory failure has been reported previously [20, 25], the method is not available for everyday clinical practice. The monitoring tools used for this purpose in the first experimental and clinical studies were highly sophisticated devices with special PCO_2_-electrodes or fiber optic sensors [26]. The device we used in the presented experimental protocol proved to be a simple, noninvasive monitor for this purpose.

According to recent studies it was suggested that even the magnitude of blood loss can be estimated by tissue capnometry, and the method may also be useful in guiding fluid resuscitation during hemorrhage. Different authors [27, 28] measured buccal PCO_2_ continuously during different severity of hemorrhagic shock in rats and found that tissue PCO_2_ monitoring was reliable in the quantitation of acute hemorrhage. Baron et al. [29] measured sublingual PCO_2_ in bleeding trauma patients and found similar results. In a porcine model of hemorrhagic shock Xu and colleagues [30] compared different volume replacement protocols based on either sublingual PCO_2_ or blood pressure. The animals monitored by sublingual PCO_2_ required smaller amount of both crystalloids and transfusion, while the microcirculation, organ functions, and survival were similar in the treatment groups. Although our experiment had different goals, the results give support to both assumptions. Loss of 50% of the circulating blood volume also increased the sublingual PCO_2_ by 50%: from *T*_0_ = 41.6 ± 8.3 to *T*_2_ = 60.1 ± 9.6 Torr. On the other hand, sublingual PCO_2_ gap increased by 5-fold; therefore it seems that for this purpose this parameter may be more sensitive than sublingual PCO_2_ on its own. We did not observe strong, significant differences in the P_SL_CO_2_ and the P_SL_CO_2_ gap values between the sham-operated and the shock groups in the partial resuscitation phase, but there were significant changes in the shock group reflecting the hemodynamic changes throughout the experiment. We suggest that it is the kinetics of P_SL_CO_2_ rather than the absolute value which deserves attention. This has to be investigated in the future. In general it is important to note that P_SL_CO_2_ or P_SL_CO_2_ gap has different role and interpretation during “rapid” or “massive” and “slow” bleeding. No one needs additional indicators during massive bleeding with severe hypotension to confirm that the patient is in trouble, and neither is there time for these measurements. Therefore sublingual capnometry may prove its merit during slow bleeding and hypovolemia as one of the potential end points of resuscitation of the microcirculation.

Massive bleeding in our study resulted in severe perfusion abnormalities as indicated by significant deterioration of sublingual CPR and RBCV, which was also reflected by changes of the sublingual P_SL_CO_2_ gap. Although the close relationship between the sublingual perfusion and PCO_2_ has already been described [6, 31], and investigations on mucosal PCO_2_ and the microcirculation of the ileum [32] have been performed in hemorrhagic shock, this is the first study to reveal a correlation between sublingual capnometry and directly measured microcirculatory parameters during hemorrhagic shock.

### 4.2. Sublingual Capnometry and Global Hemodynamics

Significant changes in MAP, HR, CI, and GEDVI were detected during the shock phase and during partial resuscitation, with the CI being significantly higher by the end of resuscitation as compared with the baseline, possibly because of the sustained tachycardia caused by the bleeding-related stress response. There are several studies showing that hemorrhage-caused hypovolemia is accompanied by sublingual hypoperfusion and/or the increase in P_SL_CO_2_ [13, 20, 29]. Nevertheless, it is important to acknowledge that P_SL_CO_2_ on its own is a poor indicator of regional circulatory changes, unless it is put in the context of the arterial and/or end-tidal PCO_2_. Alternatively, in order to eliminate the influence of global respiratory alterations, minute ventilation should be constant. This may explain why in a laboratory model of progressive hypovolemia caused by lower body negative pressure Chung et al. did not confirm the sensitivity of sublingual capnometry in the early phase of cardiovascular collapse [33]. In our opinion the main limitation of that study is that in their model minute ventilation was not constant (subjects were spontaneously breathing), end-tidal PCO_2_ decreased significantly, and they measured P_SL_CO_2_ and not P_SL_CO_2_ gap. By calculating gap values substantial P_SL_CO_2_ − P_ET_CO_2_ gap differences could have been detected. There are other important conceptual differences between their model and the earlier experimental protocols; that is, hypovolaemia was not caused by bleeding, the observation period was only 20 min, and the study population was young, healthy, nonsmoking subjects with presumably good physiologic reserves. Finally, the sublingual microcirculation was not monitored in this study, so the changes of sublingual microvascular perfusion during the experiment remain unknown.

### 4.3. Sublingual Capnometry and Oxygen Delivery/Consumption

Although the most accurate way to assess cardiac output, oxygen delivery, and consumption is invasive hemodynamic monitoring, it is often unavailable in emergencies. Simple blood gas driven variables such as ScvO_2_ and PcvaCO_2_ can help the clinician in defining the need for fluid resuscitation and red blood cell transfusion or may serve as therapeutic targets of goal-directed therapy in high-risk surgical or septic patients [34–36]. In our study ScvO_2_, O_2_ER, and PcvaCO_2_ showed significant changes during hemorrhagic shock and partial resuscitation. Although in cases of impaired oxygen uptake ScvO_2_ values can be elevated [5, 37], our hemorrhagic shock-resuscitation model gives further support to the theory that low ScvO_2_ and high PcvaCO_2_ indicate hypovolemia and they also correlated well with P_SL_CO_2_ gap values. In fact correlation of ScvO_2_ and PcvaCO_2_ with P_SL_CO_2_ gap was better than with P_SL_CO_2_, indicating that the actual condition of the microcirculation is reflected more precisely by gap values than by absolute values of sublingual PCO_2_.

## 5. Conclusions

This new capillary tonometer may be an appropriate tool for the indirect evaluation of the sublingual microcirculation. There are also some limitations to the use of this method, such as the relatively long equilibration time and the need to draw arterial blood samples to determine the P_SL_CO_2_ gap. However, the calculation of gap values is probably not necessary if the alveolar ventilation is considered stable. In our opinion, this device can be best utilized during emergency situations (in the ICU or ER and during major/high-risk surgery), where arterial and central venous catheters are commonly used, and excessive invasiveness should therefore not be a concern.

With these restrictions we concluded that capnometry-derived variables followed the microcirculatory changes and correlated with well-established indices of global hemodynamics in hypovolemia and hemorrhagic shock. Combination of these results with central venous oxygen saturation and central venous-to-arterial carbon-dioxide partial pressure differences may be complementary tools for monitoring and treating hypovolemia and hemorrhagic shock in the clinical setting.

## Supplementary Material

Figure S1. Representative examples of the sublingual microvasculature using an orthogonal polarization spectral (OPS) imaging device: (A) Normal capillary density and perfusion rate at baseline (T_0_); (B) Decreased capillary density, stop flow or sluggish intermittent capillary flow rate (non-continuous red blood cell lines in capillaries) at the end of hemorrhagic shock (T_2_); (C) Decreased capillary density with intermittent capillary flow rate at the end of partial resuscitation phase (T_6_)
