# Supplementary figures and images for: Monitoring Microcirculatory Blood Flow with a New Sublingual Tonometer in a Porcine Model of Hemorrhagic Shock
Source: Biomed Res Int. 2015 Oct 4;2015:847152. doi: 10.1155/2015/847152 (PMC4609384; doi:10.1155/2015/847152)

Figure S1.

A

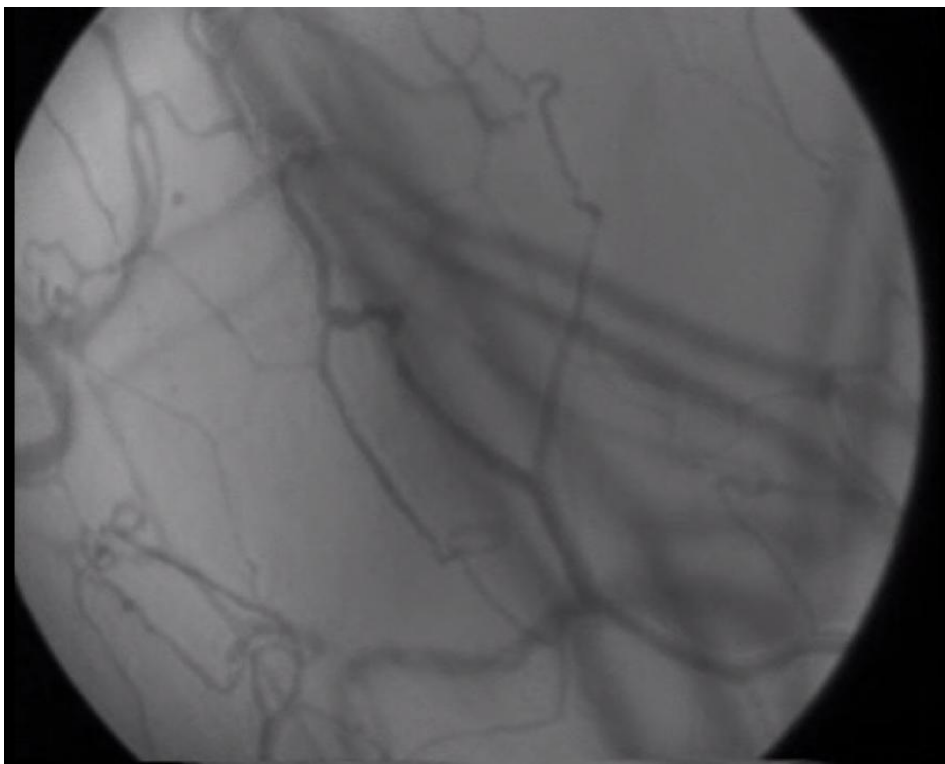

B

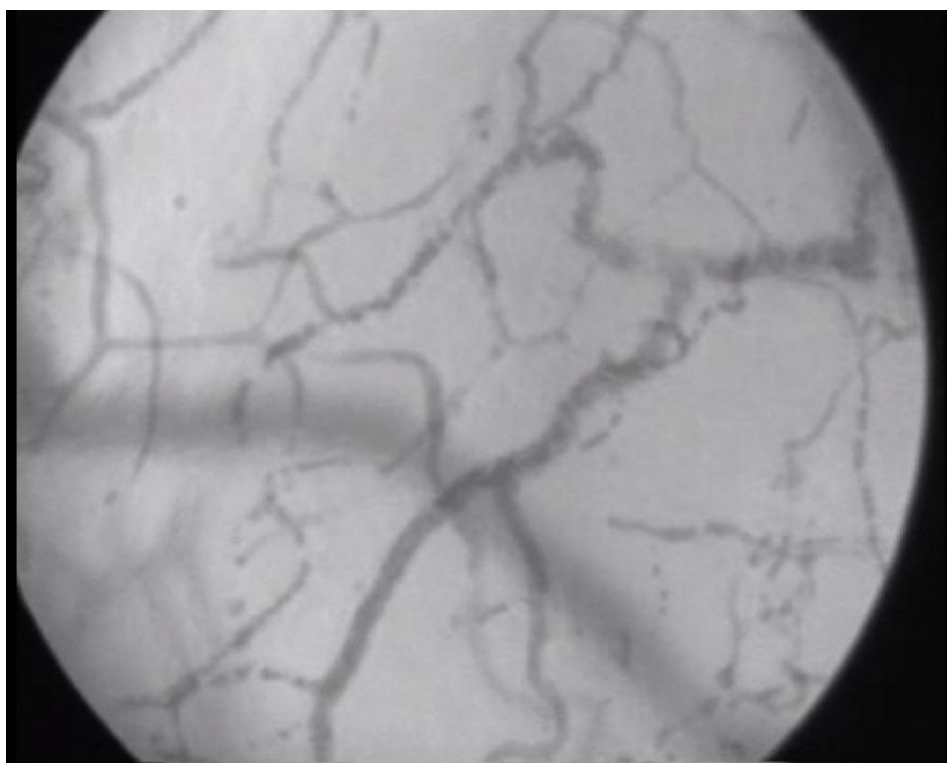

C

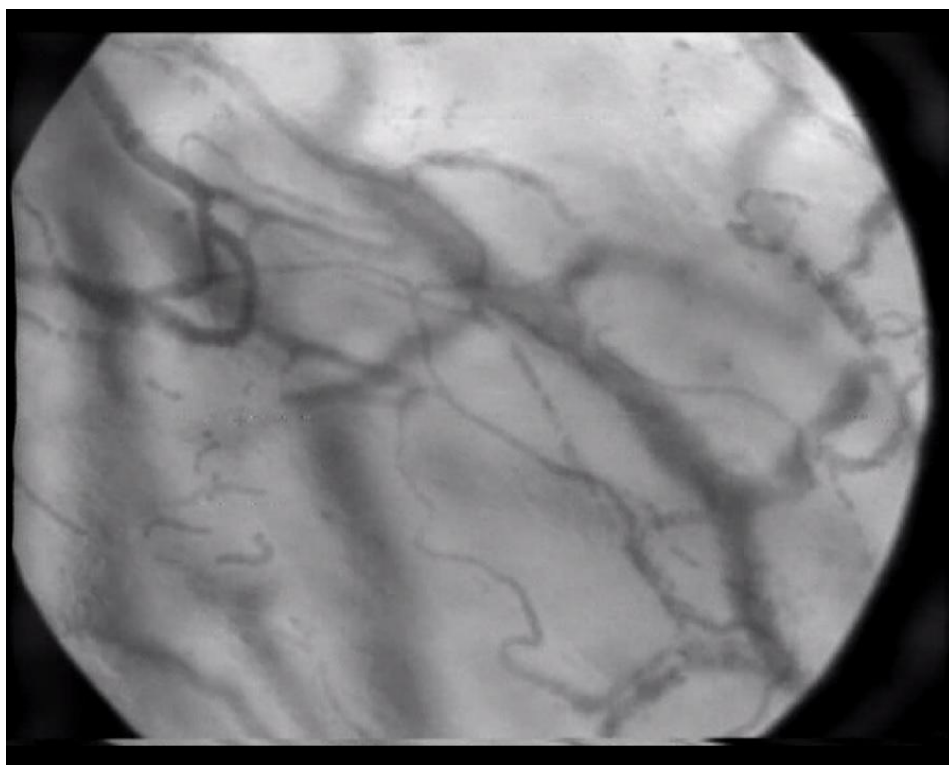

Supplement: Supplementary file 1 — Figure S1. Representative examples of the sublingual microvasculature using an orthogonal polarization spectral (OPS) imaging device: (A) Normal capillary density and perfusion rate at baseline (T0); (B) Decreased capillary density, stop flow or sluggish intermittent capillary flow rate (non-continuous red blood cell lines in capillaries) at the end of hemorrhagic shock (T2); (C) Decreased capillary density with intermittent capillary flow rate at the end of partial resuscitation phase (T6) [file 847152.f1.pdf]
